# Supplementary figures and images for: Changes in horizontal strabismus after inferior rectus muscle recession with or without nasal transposition in thyroid eye disease: A retrospective, observational study
Source: PLoS One. 2020 Oct 1;15(10):e0240019. doi: 10.1371/journal.pone.0240019 (PMC7529222; doi:10.1371/journal.pone.0240019)

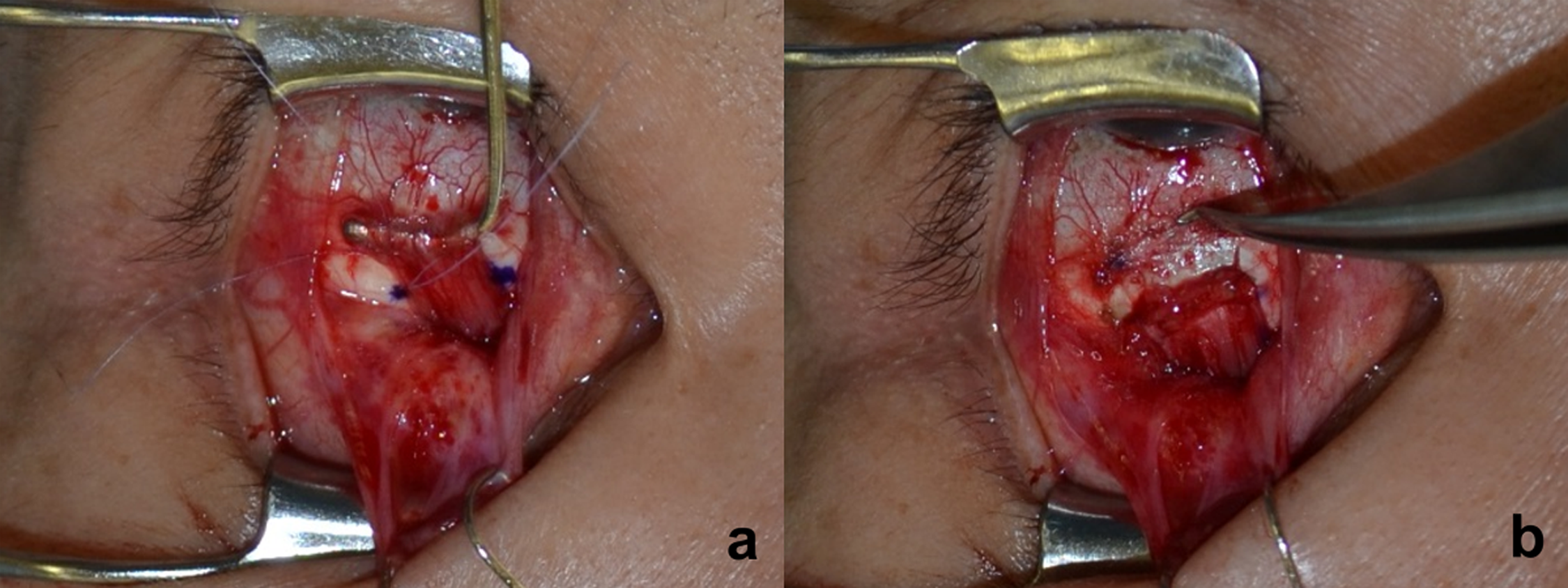

Supplement: S1 Fig — a. After securing the inferior rectus muscle at its insertion with a muscle hook, the muscle tendon is secured using locking 8–0 polyglactin sutures at two points, 1 mm posterior to the globe insertion. b. The inferior rectus muscle is recessed and fixed onto the sclera. (TIF) [file pone.0240019.s001.tif]
